# Age and hemispheric differences in transcallosal inhibition between motor cortices: an ispsilateral silent period study

**DOI:** 10.1186/1471-2202-14-62

**Published:** 2013-06-25

**Authors:** Travis Davidson, François Tremblay

**Affiliations:** 1 School of Human Kinetics, University of Ottawa, Montpetit Hall, 125 University Private, Ottawa, ON K1N 6N5, Canada; 2 School of Rehabilitation Sciences, University of Ottawa, Guindon Hall, 451Smyth Rd., Ottawa, Ontario K1H 8M5, Canada; 3 Bruyère Research Institute, 75 Bruyère St, Ottawa, Ontario K1N 5C8, Canada

## Abstract

**Background:**

In this study, we investigated age and hemispheric differences in transcallosal inhibition (TCI) in the context of active contraction using the ipsilateral silent period (iSP). We also examined whether age-related changes in TCI would be related to corresponding changes in manual performance with age. Participants consisted of right-handed individuals from two age groups (young adults, n=13; seniors, n=17). The iSP was measured for each hemisphere using suprathreshold TMS pulses delivered over the primary motor cortex ipsilateral to the maximally contracting hand while the homologue muscles of the opposite hand were lightly contracting (~15% of the maximum). Manual performance was assessed bilaterally for both grip strength and fine dexterity.

**Results:**

Our results yielded two main findings. First, TCI measures derived from iSP were strongly influenced by age, whereas differences between hemispheres were only minor. Second, correlation analyses revealed that age-related variations in TCI measures were related to changes in manual performance, so that left-to-right TCI correlated with right hand performance and vice-versa for the opposite hand/hemisphere.

**Conclusion:**

Overall, these results concur with other recent reports indicating that mutual inhibition between motor cortices tends to decline with age. In this respect, our observations are in line with the notion that the balance of normally predominantly inhibitory interactions between motor cortices is shifted toward excitatory processes with age.

## Background

Coordination of movements relies on both the activation of each independent hemisphere and the communication between hemispheres which is mediated via the corpus callosum [1]. This transcallosal pathway is essential for the interhemispheric transfer of perceptual, sensory and motor information underlying complex and integrated behaviors [2]. Although transcallosal connections can be facilitatory, mutual inhibition appears to be the primary mode of action between the two primary motor cortices [3]. This mutual inhibition has been shown to be finely modulated depending on task demands, unilateral actions leading to increased inhibitory drive from the active hemisphere, whereas bilateral actions lead to more balanced inhibition between hemispheres allowing for coordinated actions of the two extremities [4,5]. In the primary motor cortex (MI), interhemispheric inhibition can be assessed non-invasively with transcranial magnetic stimulation (TMS) using either paired-pulse paradigms or via the ipsilateral silent period (iSP) [6]. With paired-pulse paradigms, the target hemisphere is first conditioned by applying suprathreshold TMS on the opposite hemisphere at short (8–12 ms) or long (e.g., 40 ms) inter-stimulus intervals leading to two corresponding periods of inhibition of test motor evoked potentials (MEPs), i.e. short-latency (SIHI) and long-latency (LIHI) interhemispheric inhibition. The iSP assesses transcallosal inhibition by applying focal TMS to the motor cortex ispsilateral to the test hand while the target muscle is activated voluntarily, leading to a brief interruption of the ongoing muscle activity [7]. This interruption of voluntary muscle activity is thought to reflect transcallosal inhibition (TCI) mediated by the MI opposite to the one being active for maintaining the contraction [7]. As stressed by Chen et al. [8], although SIHI/LIHI and iSP both reflect interactions between motor cortices, they should be considered complementary rather that equivalent measures of TCI since their effects appear to be mediated by different neuronal populations.

While TMS investigations have highlighted the critical role of interhemispheric inhibition in the acquisition and transfer of motor skills [9], there is still limited information as to how this process changes as people advance in age. Indirect evidence of changes in interhemispheric inhibition with age comes from observations of difficulties experienced by older adults in bimanual tasks along with the presence of motor overflow during performance of unimanual actions [reviewed in [10]. This is paralleled by reports from functional neuroimaging studies showing that task-related activation patterns in sensorimotor areas are typically less lateralized and more widespread in older adults than in young adults [11]. Such changes in brain activation patterns are consistent with reports of structural alterations in the corpus callosum with age affecting its integrity in terms of the quantity and quality of white matter [12,13]. As suggested by Seidler and colleagues [10], such observations point to a shift in the balance of mutual inhibition between motor cortices with age towards excitatory processes. In agreement with this view, Talelli et al. [14] reported that the degree of LIHI from the left MI to the right MI during right hand grip was progressively reduced with advancing age, there was even a switch from inhibition to facilitation in the very old participant. Interestingly, the age-related reduction was not observed for SIHI, a differential effect the authors ascribed to putative physiological differences between SIHI and LIHI. In line with this, a recent investigation by Fling and Seidler [15] using the iSP as an index of transcallosally-mediated inhibition associated with voluntary contraction, also failed to detect age differences although young participants tended to show longer iSP durations than old participants. Thus, although observations from behavioral and neuroimaging studies seem compatible with an age-related shift in the balance of mutual inhibition between motor cortices, recent findings from TMS studies remains controversial in this regard.

Another related controversial topic with regard to hemispheric interactions pertains to laterality issues associated with manual asymmetries. Given anatomical and physiological evidence pointing to a leftward asymmetry in the organization of the hand motor representation in strong right-handers [16], one would expect that the balance of inhibition between motor cortices would favour the dominant (left) over the non dominant (right) hemisphere in most individuals. Surprisingly, very few TMS studies have examined this issue specifically. In line with the existence of a leftward asymmetry, early investigations by Netz et al. [17] showed that levels of SIHI from the dominant hemisphere was greater than that elicited from the non-dominant hemisphere in right-handers. More recent investigations, however, showed that such asymmetries in interhemispheric inhibition observed in the resting state are not necessarily present in the active state [18]; highlighting the importance of examining interhemispheric interactions in the context of voluntary contraction.

In the present study, we investigated mutual inhibition between motor cortices using the iSP as an index of transcallosally mediated inhibition in healthy young and senior adults. We first asked whether differences existed between the dominant and non dominant MI in strong right-handed participants and whether these differences were affected by age. Then, we asked whether age-related variations in the strength of TCI from one hemisphere would be related to changes in performance of the contralateral hand with age.

## Results

### Manual performance and corticomotor excitability

Right-left differences in manual performance and in basic measures of corticomotor excitability are described in Table 1 for the two groups. As expected, both young and senior participants exhibited significantly better performance in terms of dexterity and grip strength with their right dominant hand (Young, t_12_>3.75, p<0.003; Senior, t_16_>3.52, p<0.01) when compared to the left hand. It is also apparent in Table 1 that young participants clearly outperformed their senior counterparts. In terms of corticomotor excitability, both age groups showed a tendency for higher resting motor threshold (rMT) in the left hemisphere (i.e., right hand in Table 1) than in right hemisphere; a difference that reached significance only in the senior group (t_15_=3.01, p=0.008). Apart from this difference, the other paired comparisons revealed no significant right-left differences in either MEP characteristics (amplitude and latency) or in contralateral silent period (cSP) duration in both groups. Finally, as for manual performance, age differences were also clearly evident in corticomotor excitability, all measures pointing to a decrease (e.g., elevated rMTs) in the senior group. These age differences in excitability were accounted for when examining age-related variations in ispsilateral inhibition, as described in the next section.

**Table 1:** **Characteristics of the participants with respect to demographics**, **hand function and basic measures of corticomotor excitability** (**All values represent Mean** ± **SD**)

	**Young**	**Senior**
	**(n=13)**	**(n=17)**
Demographics		
Age (years)	22.4 ± 3.0	73.0 ±7.6
Gender (n)	9 M, 4 F	6 M, 11 F
Edinburgh Handedness score (/20)	15.7 ± 3.4	17.7 ± 2.6
Hand Function		
Dexterity	RH: 55.5 ± 4.9**	RH: 88.5 ± 33.6**
(GPT in s)	LH: 67.5 ± 6.9	LH: 100.3 ± 38.9
Pinch	RH: 10.1 ± 2.6**	RH: 7.2 ± 1.6
Strength (kg)	LH: 9.1 ± 2.4	LH: 6.7 ± 1.7
Corticomotor excitability ^a^		
rMT	RH: 61.0 ± 11.3	RH:70.1 ± 11.5**
(% output)	LH: 57.6 ± 8.3	LH: 65.9 ± 13.0
MEP amplitude (mV)	RH: 5.6 ± 1.8	RH: 3.6 ± 1.2
	LH: 5.5 ± 1.8	LH: 3.5 ± 1.5
MEP Latency (ms)	RH: 20.2 ± 1.8	RH: 21.8 ± 2.1
	LH: 20.1 ± 1.5	LH: 21.7 ± 2.3
cSP duration (ms)	RH: 141.5 ± 34.8	RH: 115.9 ± 24.2
	LH: 148.9 ± 37.9	LH: 115.3 ± 26.0

^a^All measures were derived from the hand contralateral to the hemisphere stimulated during the cSP/iSP assessment procedure. TMS data from one senior was incomplete (n=16).

Abbreviations: *GPT* Grooved Pegboard Test, *RH* Right Hand, *LH* Left Hand, MEP, *rMT* resting Motor Threshold, Motor Evoked Potential, *cSP* contralateral Silent Period.

**Significant right-left differences at p<0.01 in paired t-test comparisons.

### Age and hemispheric differences in ispsilateral inhibition

**Figure 1:**
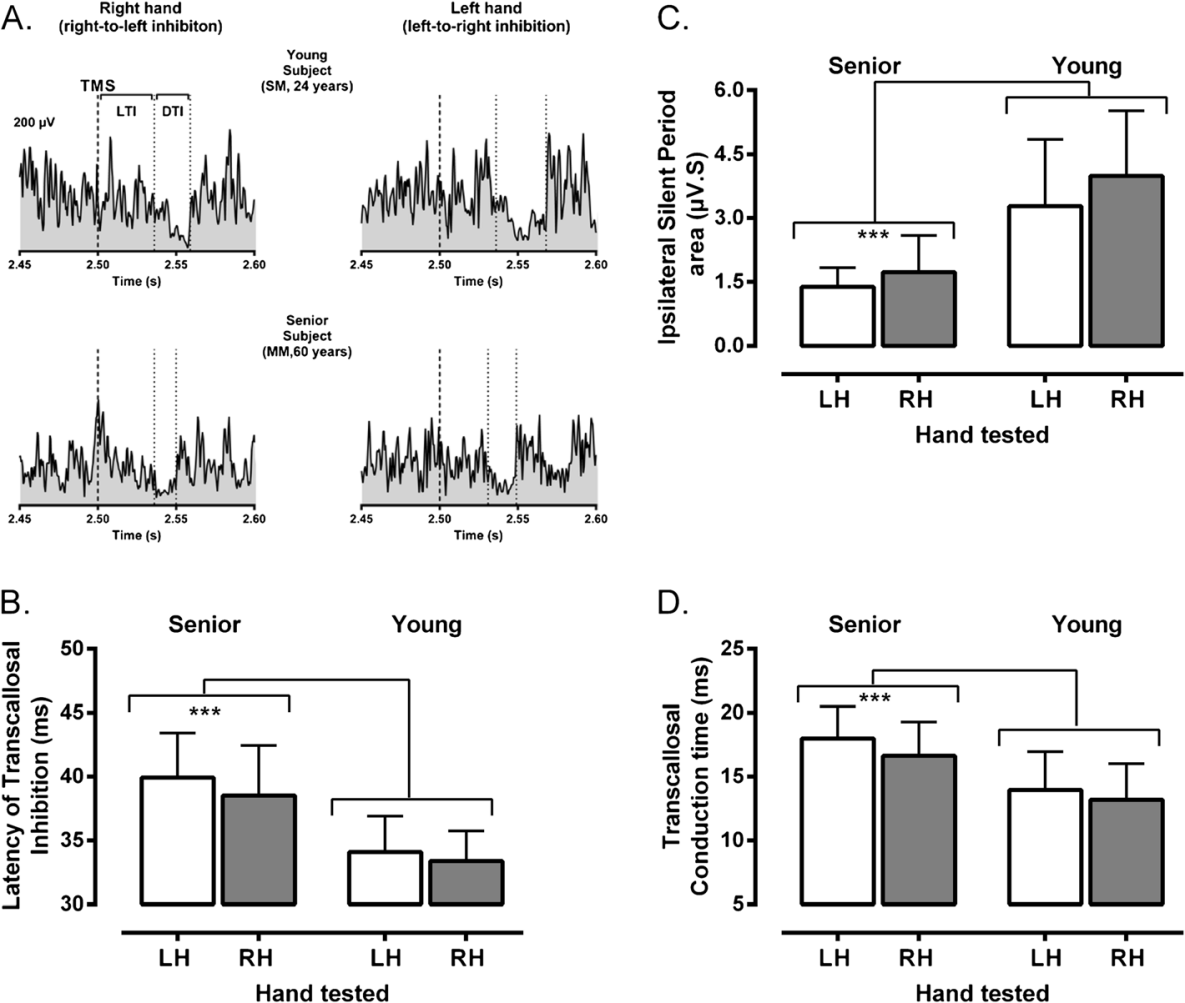
**Examples of ispsilateral inhibition and mean group differences. A**. Examples of rectified and averaged EMG traces (n=5 trials) depicting ipsilateral silent periods elicited in response to transcranial magnetic stimulation (TMS) in two participants, young and senior. As illustrated, several measures of transcallosal inhibition could be derived from iSP recordings. The latency of transcallosal inhibition (LTI) was computed as the time interval between the cortical stimulus (thick dotted line) and the iSP onset (first thin dotted line), which was determined as the first sustained decline in EMG activity when compared to mean pre-stimulus level (horizontal dotted line). The duration of transcallosal inhibition (DTI) was estimated as the time interval between the iSP onset and offset (second thin dotted line), which as determined as the point where the EMG activity returned to pre-stimulus level. Finally, the depth of ispsilateral inhibition was estimated by calculating the iSP area, as depicted by the blank area delimited by the two vertical dotted lines (DTI) and below the horizontal line in the recordings. Note that timing measurements are given only for illustrative purposes, as the real estimates were derived from a trial-by-trial analysis. **B**. **C** and **D**. Mean variations (± 1 SD) in measures of ispsilateral inhibition (**B**, LTI: Latency of transcallosal inhibition; **C**, iSP area: ispsilateral silent period area, **D**, TCT: transcallosal conduction time) derived from each hand/ hemisphere for the two groups of participants. Note again the major differences between age groups as indicated by the asterisks (p<0.001).

Variations in measures of ispsilateral inhibition with respect to age and hemisphere are illustrated in Figure 1. Note that the final analysis of iSP data excluded two senior participants. The first case had incomplete data due to poor tolerance for TMS at high intensity. The second case was an outlier, as detected with the Grubb’s test (Z=3.67, p<0.01), with very long iSPs. Two main observations can be made from inspection of Figure 1. First, only relatively small differences were observed between hemispheres in the two age groups. This is evident in Figure 1A showing examples of iSP recordings obtained from each hemisphere in a typical senior and young participants. Second, age had a major impact on TCI with seniors showing delayed onset latency of transcallosal inhibition (LTI), decreased iSP area and prolonged transcallosal conduction time (TCT). The impact of age can be easily appreciated by looking at the mean variations in LTI, iSP area and TCT measured in each age group, as illustrated in Figure 1 (B, C and D). The ANOVA confirmed that “hand/hemisphere” had little influence on iSP measures, with only a marginal trend noted for iSP area (F_1,26_=3.8, p=0.06) owing to the interhemispheric difference observed in the young group (Figure 1C). The large influence of “Age Group” on TCI measures (F_1,26_>20, p<0.001) was also confirmed, this factor alone accounting for >40% of the total variance observed in LTI, iSP area and TCT measures. For all TCI measures, no interactions were found between “hand/hemisphere” and “Age Group” (F_1,26_<1, p>0.49).

The large “Age” effect found in the primary analysis prompted a secondary analysis to better delineate the impact of this factor and also to address possible influences arising from age differences in corticomotor excitability. For this secondary analysis, all right and left TMS measures obtained in each participant were averaged to get single mean values. Then, chronological age, rMT, MEP amplitude, MEP latency and cSP duration were entered as co-variates into univariate analyses of co-variance (ANCOVA) to examine their respective impact on each measure of TCI (i.e., LTI, iSP area and TCT). This series of ANCOVAs confirmed the significant impact of chronological age (F_1,22_>4.5, p<0.05), this factor alone accounting for respectively 17%, 24% and 23% of the variance in LTI, iSP area and TCT measures. Besides a significant effect of MEP latency on LTI measures (F_1,22_=11.1, p=0.002), which was expected given that LTI depends to a large extent on MEP latency, no other co-variates had a significant effect on measures of TCI (F_1,22_<3.6, p>0.07).

### Relationships between transcallosal inhibition and manual performance

**Figure 2:**
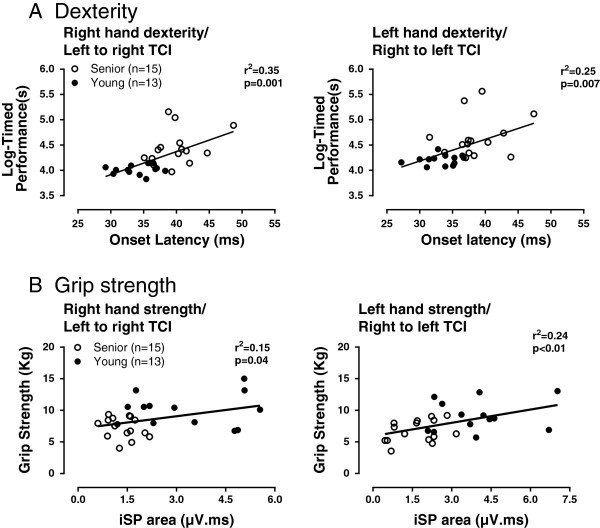
**Illustrations of relationships found between measures of transcallosal inhibition (TCI) derived from each hemisphere and age**-**related variations in unimanual performance in dexterity (A) and strength (B) tests.** In both **A** and **B**, the relationships are given with respect to the direction of TCI induced from one hemisphere to the other and performance of the contralateral hand controlled by the stimulated hemisphere; so that left to right TCI is correlated with performance of the right hand. **A**. Relationships between onset latency of TCI and contralateral dexterity, as measured with the Grooved Pegboard Test after log-transformation of timed performance. **B**. Relationships between the ipsilateral silent period area (iSP) and contralateral grip strength, as measured with a pinch dynamometer. Note that correlational analysis omitted two subjects in the senior group, one with incomplete data and one detected as an outlier.

The results of the correlation analysis examining the relationships between measures of interhemispheric inhibition and age-related variations in unimanual performance in the right and left hands are described in Table 2. As evident in the Table 2, the associations were generally stronger for the dexterity than for the grip strength test and this for both hands. The nature of these relationships can be further appreciated in Figure 2. It can be seen, for instance, that age-related variations in LTI derived from each hemisphere were strongly related to dexterity of the contralateral hand; delayed onset being associated with slower performance (Figure 2A). Likewise, age-related variations in iSP area explained a significant proportion of the variance observed in contralateral grip strength, especially for the left hand with right to left TCI (Figure 2B).

**Table 2:** **Associations between measures of transcallosal inhibition and age**-**related variations in unimanual performance of the right hand and left hand**

**Direction of motor transcallosal inhibition**^**a**^	**Manual performance**	
	**Dexterity (GPT)**	**Grip strength (Pinch dynamometer)**
Left to right	Right Hand	
LTI	*r*= 0.59***	*r*= −0.37
TCT	*r*= 0.50**	*r*= −0.46*
iSP area	*r*= −0.46*	*r*= 0.38*
Right to left	Left Hand	
LTI	*r*= 0.50**	*r*= −0.33
TCT	*r*= 0.42*	*r*= −0.44*
iSP area	*r*= −0.57**	*r*= 0.49**

^a^ The direction of transcallosal inhibition corresponds to the ispsilateral inhibition induced from the stimulated hemisphere towards the opposite hemisphere during voluntary contractions.

Abbreviations: *GPT* Grooved Pegboard Test, *LTI* Latency of Transcallosal Inhibition, *iSP* ispsilateral Silent Period area, *TCT* Transcallosal Conduction Time.

Significant correlations are marked with asterisks: *p<0.05, **p<0.01, ***p<0.001.

## Discussion

In the present study, we investigated age and hemispheric differences in mutual inhibition between motor cortices during voluntary activation using the iSP to probe TCI. Two main findings emerge from our observations. First, TCI indices derived from iSP characteristics (i.e., LTI, iSP area and TCT) were strongly influenced by age differences, whereas differences between hemispheres were only marginal. Second, correlation analyses revealed significant relationships between indices of TCI derived from each hemisphere and performance of the contralateral hand in dexterity and grip strength tests, so that left-to-right TCI correlated with right hand performance and vice-versa for right-to-left TCI and left hand performance. In the following discussion, we address the significance of these findings for the study of aging and its impact on motor systems.

### Age and hemispheric differences in TCI

Contrary to evidence suggesting an asymmetry in the balance of mutual inhibition in favor of the dominant MI in right-handed individuals [17,19,20], only minor differences in TCI were found between the two hemispheres in our two groups of participants. In this respect, our results appear consistent with those of De Gennaro et al. [3], who found no difference in interhemispheric inhibition between the two hemispheres in resting state using bifocal TMS in young adults. Our observations for the senior group are also in line with those of Lewis & Perrault [18] who also failed to detect differences in paired-pulse SIHI between the dominant and non dominant hemisphere in their group of healthy senior adults, who served as controls for stroke patients. Interestingly, the absence of hemispheric asymmetry reported in Lewis & Perrault’s study was evident only when the target hand (contralateral to the test hemisphere) was active, irrespective of whether the ipsilateral hand was active or not. In the present study, the fact that iSP was tested during concurrent contraction of both hands might have been critical in attenuating possible asymmetries in the level of mutual inhibition in relation to manual dominance. It remains that the issue of hemispheric asymmetries in relation to manual dominance remains a controversial topic in the TMS literature [e.g., see [21] and more research is needed with larger groups of right- and left-handers to address the question.

Contrasting with the relatively minor difference found between hemispheres, a major difference was found between age groups, senior participants showing delayed LTI, prolonged TCT and reduced iSP area when compared to young participants. Given that our group of seniors exhibited signs of decreased corticomotor excitability, it is possible that the observed age difference in TCI might have reflected impaired motor activation or peripheral nerve dysfunction. This possibility seems unlikely, however, for several reasons. First, with regard to motor activation, the work of Giovannelli et al. [22] has clearly demonstrated that TMS-induced ispsilateral inhibition is little affected by the intensity of contraction in the test hand, the most important factor being rather the presence of light activity in the opposite hand. During testing, all our participants, and especially seniors, were encouraged to produce their maximum effort in the ispsilateral hand, while lightly contracting the other hand. Thus, by basing our assessment on Giovannelli’s method, we made sure that conditions would be optimal to elicit ipsilateral inhibition in each age group, in spite of individual variations in muscle activation. Second, as demonstrated by our secondary analysis (ANCOVAs), age-related variations in various indices of corticomotor excitability had little influence on measures of ispsilateral inhibition. In fact, the only significant interaction found was between LTI and contralateral MEP latency, which was expected. However, the computation of TCT, which largely removes the influence of MEP latency, showed that age differences were still highly significant. To summarize, while our seniors exhibited typical signs of corticomotor aging [e.g., [23], these changes could hardly account for the large age effect observed on iSP measures; pointing to central alterations in TCI as the primary cause.

In fact, our observations appear to be congruent with the proposal of Seidler’s group [10] that there is a shift with age in the balance of mutual inhibition between motor cortices towards excitatory processes. However, as noted earlier, these investigators [15] found only a trend when comparing the strength of ispsilateral inhibition in young and old adults. Their observation that young adults tended to show deeper levels of ipsilateral inhibition than older adults seems consistent with the age difference reported here. In more direct line with our results, Boudrias et al. [24] observed a strong age effect when comparing measures of interhemispheric inhibition derived from bi-focal TMS in young and older adults. In their study, a clear distinction was seen between young and older adults in that most seniors showed evidence of transcallosal facilitation rather than the typical inhibition. In the same vein, McGregor et al. [25] found that measures of ispsilateral inhibition were significantly reduced in older subjects when compared to young. Interestingly, the largest reductions were seen in the group of sedentary seniors, where iSP durations were on average 50% shorter than in young adults; a range comparable to the averaged reduction in iSP area reported here (57%). In physically active seniors, the reduced ipsilateral inhibition was less pronounced (~25%), which led McGregor et al. to conclude that engaging in regular physical activity could help to maintain levels of interhemispheric inhibition. Although we did not specifically control for activity level in our study, it is worth noting that the outlier senior who showed exceptionally long iSP duration (see Results) was also highly active as judged by self-report. It would be interesting for future studies to investigate how interactions between advancing age and levels of physical activity influence hemispheric interactions, but the small sample size used in the present study precludes any conclusion in this regard. Nevertheless, both the present findings and the results of recent TMS studies concur with the notion that transcallosally mediated inhibition becomes less efficient with age in line with reports describing structural alterations in the integrity of callosal fibres between motor cortices with advancing age [15,24,26].

Regarding the physiological mechanisms underlying the observed changes in iSP measures with age, the reduced iSP area points to a decrease in the excitability of ispsilateral transcallosal inhibitory neurons. Such a decrease would be consistent with reports of age-related reductions in the excitability of local inhibitory circuits mediating short-interval intra-cortical inhibition (SICI) [27] and short-latency afferent inhibition [28] reported in the motor cortex of seniors. As shown by Avenzino et al. [29], local interneurons involved in transcallosally mediated inhibition share common properties with those controlling excitability of pyramidal tract neurons; and thus, any alterations in intra-cortical excitability with age could also affect inhibitory connections between motor cortices. In parallel, the delayed LTI and prolonged TCT found in seniors are consistent with reports of structural alterations in callosal connectivity between motor cortices with age, as stated earlier. In fact, a growing body of evidence [12,30] is now emerging linking preserved task-related functional connectivity between hemispheres in seniors with integrity of transcallosal connections. Indeed, as suggested by our correlational analysis, integrity of transcallosal connections seems to be important in allowing older adults to maintain certain levels of performance, as discussed below.

### Relationship between ipsilateral inhibition and manual performance

The result of our correlation analysis revealed significant relationships between our different measures of TCI in each hemisphere and performance of the contralateral hand, so that left to right TCI (left hemisphere stimulated) correlated with right hand performance and vice versa for the left hand. Interestingly, these associations were particularly strong for the dexterity task, which is consistent with the purported role of TCI in preventing motor overflow when task demands require fine unilateral control of one hand [31]. While correlations with grip strength were not as strong, good quality relationships were still found, for example, between iSP area and left hand; suggesting a role of TCI in unimanual force production. In line with this, Fling and Seidler [4] recently reported an inverse relationship between measures of ispsilateral inhibition and the ability of young individuals to suppress motor activity in the resting hand (i.e., motor overflow) during a unimanual force production task. Although we did not monitor motor activity in the resting hand during our tests of manual performance, our observations on the association between measures of TCI and manual performance are consistent with those of Fling and Seidler with regard to the role of TCI in allowing fine independent control of unimanual performance. In a related study from the same group of investigators [13], the association between functional motor activation and performance of a precision task with the dominant hand was examined in young and old adults. Much like in the present study, the authors found that increased ispsilateral motor recruitment (and presumably less efficient TCI) was associated with poorer task performance. The fact that this association was found only for the older group and not in younger subjects does not invalidate the comparison with the present findings since both their results and ours converge to show that proper levels of TCI is an important factor in leading to fine motor performance in the context of precision tasks, especially as people advance in age. In fact, there is ample evidence from TMS studies that levels of intra- and interhemispheric inhibition are critical for the performance of fine motor tasks [14,32-34]. With regard to aging specifically, the observation that our group of seniors exhibited various levels of impaired ispsilateral inhibition and that these impairments were in part reflected in their dexterity performance would be consistent with other studies in which deterioration in motor performance and in inter-limb coordination with age was associated with a decreased ability to modulate inhibition at the central level [14,34-36].

## Conclusion

The present study examined age and hemispheric differences in mutual inhibition between motor cortices using the iSP as a marker of transcallosally mediated inhibition. Consistent with previous studies, we report a major difference with regard to age, whereas differences between hemispheres were only marginal. In addition, we show that measures of TCI derived from each hemisphere correlated well with age-related variations in manual performance of the contralateral hand. Overall, these results appear congruent with the hypothesis proposed by Seidler and colleagues [10] suggesting a shift with age in the overall balance of normally predominantly inhibitory interhemispheric interactions toward excitatory processes. Possible limitations of the present study include the small number of participants in each age group and the fact that older participants were considered as active seniors, which may not be representative of the population of seniors in general.

## Methods

The study procedures were approved by the Research Ethics Board at the Bruyère Research Institute, Ottawa, Ontario, Canada. Written informed consent was obtained prior to participation from all participants in accordance with the *Declaration of Helsinki*. All assessments were performed in a controlled laboratory environment. Each participant received a small honorarium for his or her participation.

### Participants

Two groups of participants, young and senior were recruited for this study. The young group (n=13) was recruited from the student population at University of Ottawa, whereas the senior group (n=17) was recruited from the community in the Ottawa-Gatineau area. All participants were right-handed, as determined by the Edinburg Handedness Inventory [37]. Prior to the experimental session, all participants completed a medical questionnaire to determine their general health status and to ensure that there were no contra-indications to TMS or antecedents of conditions likely to affect their performance in the tests. In addition, sensory function of the hand was assessed using a Rydel-Seiffer tuning fork to rule out the presence of undiagnosed neuropathies. All participants exhibited vibration thresholds in line with their norms for their age range [38]. The demographic characteristics of the participants are listed in Table 1.

### Manual performance: Grooved Pegboard Test and pinch strength

For manual performance, participants were comfortably seated in front of a table. All tests were applied bilaterally and administered by the same experimenter (TD). The order of testing with each hand was determined randomly before the testing session. Manual dexterity was assessed with the grooved pegboard test (GPT, Lafayette Instr, IN 47903), which consists of inserting pegs into keyholes in a specific order as fast as possible. After instructions and practice trials, the GPT was administered once to each hand and performance was recorded as the time in seconds to complete the task. The second test consisted of grip strength assessment. For this test, a small pinch gauge (PG-60, B & L Engineering, Santa Ana, CA 92705) was used to assess the thumb-index finger pinch strength. This test also provided an index of the muscle activity elicited by the first dorsal interosseous muscles (FDI) during maximal voluntary contraction. Participants were presented with the gauge and were asked to press as hard as they can for the duration of a tone, which lasted 3 s. Three trials were performed for each hand with a 60 s rest between trials. The average provided a measure of pinch strength and of maximal activation in the FDI. To avoid any interference associated with fatigue, the strength test was always performed at the end of the testing session, after neurophysiological testing was completed.

### Electromyographic recordings

Electromyographic (EMG) activity was recorded using 10-mm auto-adhesive surfaces electrodes (Ag/AgCl, Kendall Medi-Trace™ 130) placed over the FDI muscles of each hand using a tendon-belly montage. EMG signals were amplified and filtered with a time constant of 10 ms and a low-pass filter of 1 kHz (AB-621G Bioelectric amplifier, Nihon-Kohden Corp., CA 92610). Signals were digitized at rate of 2 kHz (BNC-2090, National Instrument Corp.) and further relayed to a laboratory computer running custom software to control acquisition.

### Transcranial magnetic stimulation and resting motor threshold (rMT)

Magnetic stimulation was delivered via a Rapid^2^ stimulator (Magstim Co. Dyfed, UK) connected to a figure-eight coil (90 mm outer loop diameter). All testing was performed with the participants comfortably seated in a recording chair. Participants were fitted with a Waveguard TMS compatible cap (ANT North America Inc, WI 53719) to allow for consistent coil placement. A U-shaped neck cushion was also used to restrain head movements and prevent neck fatigue. TMS testing sessions began by first determining the “hotspot” for the FDI and then by determining the rMT for the stimulated hemisphere. This procedure was performed sequentially on each hemisphere with the order of testing between the two alternating between participants. To determine the optimal site to evoke MEP’s in the contralateral target muscle (FDI), the approximate location of the hand motor area on the stimulated hemisphere was explored in 1-cm steps until reliable MEP’s could be evoked. This site was then marked with a red dot to ensure consistent coil positioning. After localization of this stimulation “hotspot”, the rMT was determined using the procedure described by Mills and Nithi [39]. Starting from suprathreshold intensity, the stimulation intensity was gradually decreased in 1% steps until no MEP’s could be evoked in 10/10 consecutive trials (lower threshold). Then, the intensity was increased in 1% increments to find the minimal intensity that would produce reliable peak-to-peak amplitude MEPs of at least 50 μV for 10/10 consecutive trials (upper threshold). The rMT was defined for each participant as the median intensity between the upper and lower threshold values. EMG was continuously monitored on an oscilloscope, at high gain (0.1 mV/div), to ensure the absence of any muscle activity during the procedure. As mentioned above, the same procedures were repeated for the opposite hemisphere.

### Contralateral and ipsilateral silent period (cSP/ iSP)

**Figure 3:**
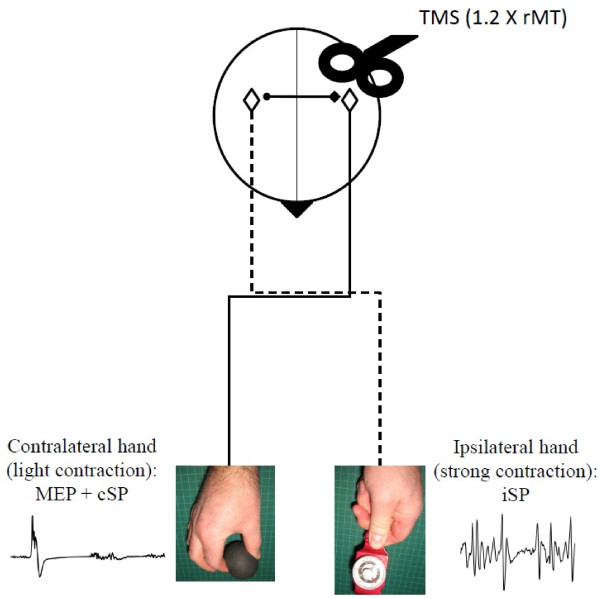
**Schematic representation of the testing paradigm used to assess the contralateral and ispsilateral silent period (cSP/iSP).** In the illustration, the iSP is elicited in the left hand during maximal effort in response to left transcranial magnetic stimulation (TMS), while the cSP with the associated motor evoked potential (MEP) is elicited in the right hand during light effort. (rMT: resting motor threshold).

The cSP and iSP were measured concurrently using the approach described by Giovannelli et al. [22], which involved maximal contraction of the hand ispsilateral to the stimulated hemisphere coupled with light contraction of the contralateral hand. As shown by these authors, voluntary activation of the contralateral hand, irrespective of the level of contraction significantly prolonges the iSP in the test hand when compared to rest and this, without affecting the level of background EMG in the ispsilateral hand. For cSP/iSP testing, participants were instructed to press as hard as they could on the pinch dynamometer using the thumb and index fingers with one hand, while lightly squeezing a soft exercise ball with their opposite hand. The latter contraction corresponded, on average, to ~15% of the maximal voluntary contraction in both age groups. Participants were trained to maintain the contractions for 3 s in sync with a tone (550 Hz) generated by the computer. The subjects were told to focus on the maximally contracting hand ipsilateral to the stimulation and were given verbal feedback to maintain contraction. In each trial, TMS was delivered on the hemisphere ipsilateral to the maximally contracting hand at 2 s in the course of the trial using an intensity equivalent to 120% of the rMT. The inter-trial interval was 10–15 s. Short pauses were allowed to prevent fatigue. Five trials were performed on each hemisphere, the order of testing between hemispheres alternating between participants. A schematic representation of the testing paradigm for the cSP/iSP assessment is provided in Figure 3.

### TMS data analysis

Data from the cSP/iSP trials were analyzed off-line from visual inspection of stored EMG traces using custom software. All visual analyses were performed by the same investigator (TD) using numerically coded files, which were saved separately from the demographic data, to allow for blinding with respect to the age of the participant. Visual analysis of silent periods has been shown to have good inter and intra-rater reliability [40,41]. For cSP measures, EMG traces obtained from the contralateral hand were examined individually to determine the duration of the SP and measure the characteristics of the associated MEP in terms of amplitude (peak to peak) and latency. The cSP duration was determined as the time from the MEP onset till the first sign of EMG recovery. Mean values were computed by averaging individual trials. The same approach was used to analyze iSP trials using EMG traces obtained from the ispsilateral hand. For iSP measures, each individual file was analysed twice with at least one week interval between runs to ensure intra-rater reliability. In each file, two main indices of TCI were derived from the EMG trace of the ipsilateral hand. First, to get an index of the interhemispheric transfer time, the onset latency of transcallosally mediated inhibition (LTI) was measured. The LTI was defined as the time interval from the cortical stimulus until the 1^st^ sign of sustained decline (>25% of mean EMG level for at least 5 ms) in the EMG activity level. Maximal and mean EMG levels were measured 100 ms prior to stimulation. The second index consisted of estimating the depth of ispsilaterally-induced inhibition by computing the iSP area. The latter was determined by first rectifying the EMG trace and then by computing the integral of the area delimited by the iSP onset and offset. As stated above, the iSP onset was determined as the first time point where the signal of the EMG activity clearly fell under the mean level observed before the cortical stimulus. The iSP offset was determined as the first time point after iSP onset at which the EMG level returned to the mean level. The reliability analysis showed a strong to very strong level of agreement, as reflected in intra-class correlation coefficients, between the two set of measurements for both the LTI (right hand, 0.93; left hand, 0.97) and iSP area measures (right hand, 0.82; left hand, 0.80). Individual examples of EMG traces analysed to derive iSP measurements are shown in Figure 1A for a participant in each age group. Finally, as a co-result of this analysis, we derived a third index of TCI by computing the transcallosal conduction time (TCT), i.e., the duration of the stimulus transfer to one hemisphere to the other, by subtracting the MEP latency obtained from the contralateral hand from the iSP onset latency (i.e., LTI, see Figure 3).

The statistical analysis was performed in three steps. First, right-left differences in manual performance and in basic measures of corticomotor excitability were examined in each age group with paired t-tests. For these paired comparisons, the significance level was set at P<0.01 to reduce the risk of type I error owing to multiple comparisons. The second step consisted of examining variations in each index of TCI (LTI, iSP area and TCT, respectively) using analyses of variance (ANOVA) for repeated measures with “Hand/Hemisphere” as the within-subject factor and “Age Group” as the between-subjects factors. For this analysis, the significance level was set at P<0.05 to detect main effects and interactions. The final step consisted of examining relationships between manual performance and measures of TCI using Pearson’s correlation. For these analyses, timed performance for the dexterity test (i.e., GPT) was log transformed to normalize the distribution which was skewed owing to the presence of participants in the senior group (4/17) with very slow performance (>120 s). The significance level for the correlations was set at P<0.05. All statistical tests were performed using SPSS software version 17.0 for Windows^®^ (Chicago, IL, USA). Figures were prepared using GraphPad Prism version 5.00 for Windows (GraphPad Software, San Diego California USA, http://www.graphpad.com).

## Abbreviations

ANOVA: Analyses of variance; DTI: Duration of transcallosal inhibition; EHI: Edinburgh inventory index; EMG: Electromyography; FDI: First dorsal interosseous; GPT: Grooved pegboard test; iSP: Ipsilateral silent period; iSP area: Area under the curve during the ipsilateral silent period; LH: Left hand; LIHI: Long-latency interhemispheric inhibition; LTI: Latency of transcallosal inhibition; MEP: Motor evoked potential; MI: Primary motor cortex; RH: Right Hand; rMT: Resting motor threshold; SIHI: Short-latency interhemispheric inhibition; TCI: Transcallosal inhibition; TCT: Transcallosal conduction time; TMS: Transcranial magnetic stimulation.

## Competing interests

The authors declare that they have no competing interests.

## Authors’ contributions

TD assisted with the study design, carried out the data collection, analyzed all data, and drafted the manuscript. FT conceived the study, aided with data collection and in drafting and editing of the manuscript. All authors read and approved the final manuscript.
